# Helicopter Control Energy Reduction Using Moving Horizontal Tail

**DOI:** 10.1155/2015/523914

**Published:** 2015-06-09

**Authors:** Tugrul Oktay, Firat Sal

**Affiliations:** College of Aviation, Erciyes University, 38039 Kayseri, Turkey

## Abstract

Helicopter moving horizontal tail (i.e., MHT) strategy is applied in order to save helicopter flight control system (i.e., FCS) energy. For this intention complex, physics-based, control-oriented nonlinear helicopter models are used. Equations of MHT are integrated into these models and they are together linearized around straight level flight condition. A specific variance constrained control strategy, namely, output variance constrained Control (i.e., OVC) is utilized for helicopter FCS. Control energy savings due to this MHT idea with respect to a conventional helicopter are calculated. Parameters of helicopter FCS and dimensions of MHT are simultaneously optimized using a stochastic optimization method, namely, simultaneous perturbation stochastic approximation (i.e., SPSA). In order to observe improvement in behaviors of classical controls closed loop analyses are done.

## 1. Introduction

Traditionally in order to control helicopters, collective and cyclic (i.e., longitudinal and lateral) rotor blade pitches are used. Presently almost all of the helicopters employ a swashplate mechanism and pitch links (it consists of two circular plates and a ball bearing arrangement separating them; see [1] for more details) to transmit two cyclic and collective pitch commands to the blade root. However, this mechanism is heavy and complex and also causes important drag during high flight speeds. Throughout history some other control methods have been considered in order to avoid these drawbacks and also for some other reasons such as reduction of control energy and redundancy in case of failures. Some of these alternatives are using trailing edge flaps (TEFs) with (see [2–4]) and without (see [5–7]) classical swashplate mechanism, passive (see [8–10]) and active (see [11–13]) helicopter morphing, and MHT (see [14–18]). For example, in [2] TEFs were integrated into blades for the case of a failure of the pitch link making the blade free float in pitch. By this method catastrophic results of a pitch link failure were corrected. In [5] TEFs were replaced with a conventional swashplate mechanism. Via eliminating swashplate mechanism and using TEFs, important reduction in weight, drag, and cost and also improvement in rotor performance were obtained. Moreover, in [8] passive morphing was used in order to reduce helicopter FCS energy. In that study many blade parameters (e.g., blade length and blade chord length) were simultaneously optimized with helicopter FCS parameters in order to save FCS energy. Substantial reduction in helicopter FCS energy was obtained using passive morphing idea. In [11] active morphing was used to save helicopter FCS energy. In that study actively morphing parameters were blade chord length, blade length, blade twist, and main rotor angular speed. The main difference between this and previous study (i.e., passive morphing) was that for the active case the helicopter design parameters are able to change (except helicopter FCS) during flight, but in prescribed interval. Using active morphing idea significant reduction in helicopter FCS energy was obtained.

MHT idea was firstly studied in [14] in 1953. In this study differential control of each side of a canted horizontal tail was permitted. In [15] collective control of horizontal tail was mechanically achieved. In this study it was claimed that a fixed horizontal tail is advantageous in order to improve longitudinal stability of helicopters in forward flight, but it is not enough during gliding and climbing flights. More recently in [16] a moveable horizontal tail to give the desired attitude at different flight speed for UH-60 was designed. Recently in [17, 18] a moveable horizontal tail was designed in order to reduce TEF deflection for swashplateless helicopters since the stroke capacity of existing smart material actuators is not enough for the required TEFs inputs. Using MHT TEFs deflections were relieved.

Numerous helicopter FCS design methods have been studied throughout the years, in historical sequence classical pole placement techniques (see [19, 20]), simple feedback control methods (see [21, 22]), and modern control approaches depend on linear matrix algebra such as linear quadratic regulator (LQR) and linear quadratic Gaussian (LQG) techniques (see [23, 24]), *H*_*∞*_ control synthesis (see [25, 26]), and model predictive control (MPC) (see [27, 28]). In this paper, a modern constrained control method, namely, OVC, is chosen for the design of helicopter FCS. OVC has many advantages with respect to the other control strategies existing in the literature. First, these controllers are modified LQG controllers and they benefit from Kalman filters as state estimators. Second, variance constrained controllers apply second-order information (i.e., state covariance matrix; see [29, 30] for details) and this kind of information is very beneficial during multivariable control system design because all stabilizing controllers are parameterized in relation to the physically meaningful state covariance matrix. Last, for large and strongly coupled multi-input, multi-output (MIMO) systems as in air vehicles control and especially in this paper, variance constrained control methods give guarantees on the transient behavior of independent variables by enforcing upper limits on the variance of these variables.

Variance constrained controllers have been used for many aerospace vehicles (e.g., helicopters, see [8, 11, 31–36]; tiltrotor aircraft, see [37]; Hubble space telescope, see [38]; tensegrity structures, see [39]) in last thirty years. For instance, in [32] variance constrained controllers were used for helicopter FCS during maneuvers, specifically level banked turn and helical turn. In that paper, performance of them was also considered during failures of some helicopter sensors. Reasonable consequences (meaning that variance constraints on outputs/inputs were satisfied and also closed loop systems were exponentially stabilized) were found in terms of helicopter FCS. Robustness of the closed loop systems (obtained via integration of linearized helicopter model and FCS) with respect to some modeling uncertainties (i.e., variation of flight conditions and all helicopter inertial parameters) was also studied and it was found that these controllers have stability robustness with respect to modeling uncertainties.

In this paper, MHT is for the first time simultaneously designed with helicopter FCS. For this purpose, a specific variance constrained controller OVC is also used for the first time for FCS. It is important to note that when MHT is integrated with classical helicopter, the number of controls increases. This causes an important result. The number of trim unknowns increases with additional MHT controls. Nevertheless, there are no additional trim equations. Therefore, in order to solve the resulting nonlinear trim equations, a useful optimization algorithm is required. For its solution, a stochastic optimization method specifically simultaneous perturbation stochastic approximation (i.e., SPSA) (see [40, 41] for brief description of SPSA) is for the first time applied for the simultaneous trimming and FCS design problem since it is computationally cheap and effective during solving constrained optimization problems when it is impossible to compute derivatives such as gradients and Hessians, analytically as in the situation herein. This paper first presents helicopter models used for simultaneous MHT and FCS design. Second, MHT is illustrated and motions of it are described. Then, definition of applied FCS (i.e., OVC) is given briefly. After that, trimming the system (i.e., the one obtained via integration of helicopter, MHT, and FCS) via simultaneous trimming and FCS design idea is explained. Then, the specific optimization method, namely, SPSA, applied in order to trim the system is summarized. Finally, this simultaneous design idea is applied for Puma SA 330 helicopter and closed loop responses of classical helicopter and helicopter with MHT are compared.

## 2. Helicopter Model

The modeling approach of used helicopter models in this paper is presented in detail in [31, 42]. The essential modeling assumptions are given next. First of all, multibody system approach was used to include all helicopter components: fuselage, horizontal tail, tail rotor hub and shaft, landing gear, and fully articulated main rotor with 4 rigid blades with blade flapping and lagging hinges. Secondly, a static inflow formulation (i.e., Pitt-Peters formulation) was applied for helicopter main rotor downwash. Thirdly, linear incompressible aerodynamics was used for the main rotor blades, but an analytical formulation was applied for the modeling of fuselage.

The modeling procedure requires using physics principles and because of the assumptions described in the previous paragraph it directly led to helicopter dynamic models that consisted of finite sets of ordinary differential equations (ODEs). This mathematical structure is fairly beneficial for control system design since it assists the direct use of modern control theory, which relies on state space representations of the system's dynamics, easily obtained from ODEs.

The modeling methodology summarized above was applied in Maple and it led to a nonlinear helicopter model in implicit form:

(1) $$\begin{array}{c} f\left({\dot{x}}_{n},x_{n},u_{n}\right)=0, \end{array}$$

where *f* ∈ *ℝ* ^28^, *x*_*n*_ ∈ *ℝ* ^25^, and *u*_*n*_ ∈ *ℝ* ^4^. Here *x*_*n*_ and *u*_*n*_ are nonlinear state and control vectors, respectively, and *ℝ* ^∗^ represents the linear space of ∗-dimensional real vectors, where “∗” can be 28, 25, or 4. It should be noted that the inconsistency between the size of *f*(28) and the size of *x*_*n*_(25) is due to the three static downwash equations. The 28 nonlinear equations in (1) are categorized as follows: 9 fuselage equations, 8 blade flapping and 8 blade lead-lagging equations, and 3 static main rotor downwash equations. The helicopter models obtained have too many terms, making its use in fast computation impractical. For that reason, a systematic model simplification technique, named ordering scheme, was applied to reduce the number of terms in the nonlinear ODEs. The ordering scheme iteratively deletes terms from an equation depending on their relative magnitude with respect to the other terms in that equation. Each term's magnitude is guessed depends on expected values that the state and control variables can take during helicopter flight (see [31, 42]). It is significant to note that the ordering scheme does not change the number or type of equations generated using physics principles; it just shortens the equations by retaining the dominant terms.

The model found after using the ordering scheme is still reasonably complex (i.e., with a total of 28 nonlinear equations). In this paper, for FCS design the nominal trajectories considered are straight level flights. When the straight level flight conditions were applied for the nonlinear equations of motion, 17 trim equations were found (i.e., 0 = 0 equations were deleted). These equations were solved using MATLAB for different straight level flight speeds. After trimming, the model was linearized using Maple, yielding continuous linear time-invariant (LTI) systems:

(2) $$\begin{array}{c} {\dot{x}}_{p}=A_{p}x_{p}+B_{p}u_{p}. \end{array}$$

Here *x*_*p*_ and *u*_*p*_ are the perturbed state and perturbed control vectors. Matrices *A*_*p*_ and *B*_*p*_ are of size 25 × 25 and 25 × 4. The state vector consists of 9 fuselage states, 8 blade flapping states, and 8 blade lead-lagging states. The control vector includes 3 main rotor controls (collective, *θ*_0_, longitudinal cyclic, *θ*_*c*_, and lateral cyclic, *θ*_*s*_, blade pitch angles) and 1 tail rotor control (collective, *θ*_*T*_).

**Figure 1:**
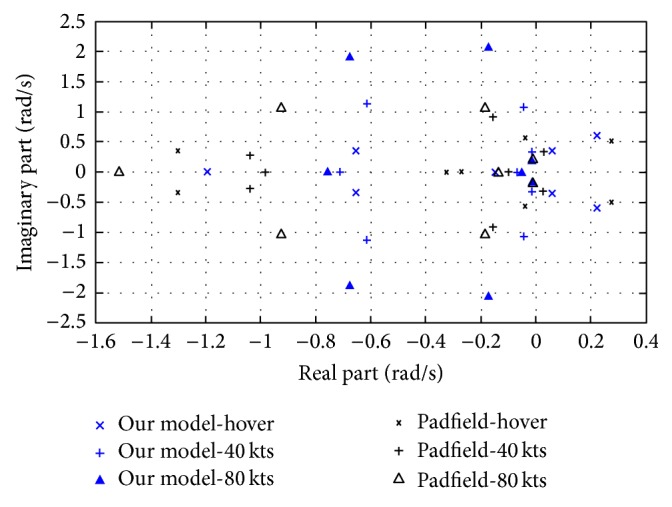
Loci of flight dynamics modes.

Puma SA 330 helicopter (see [31, 43]) was used to validate the models used in this paper. These models are leading to acceptable agreement on trim values, flight dynamics modes, and qualitatively similar flapping and lead-lagging mode behavior (see [43]). In Table 1 and Figure 1 some validation results show how the models correctly capture the dynamics of Puma SA 330 helicopter (see [31] for more validation data). For instance, most of the flight dynamics modes (linearized system eigenvalues) of the models for hover and straight level flights (i.e., 40 kts and 80 kts) match well the results reported in [43]. The mode displaying the largest discrepancy is the 4th mode (it is important to note that this is due to modeling discrepancy between the models used and [43]); nevertheless, the qualitative behavior is similar (they are both exponentially stable modes).

**Figure 2:**
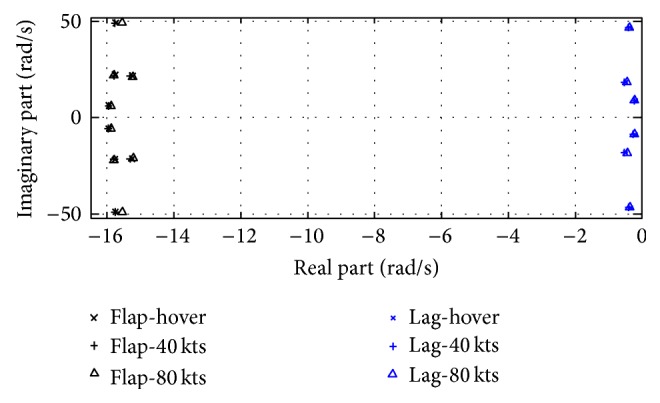
Loci of flapping and lead-lagging modes.

The qualitative behaviors of the blade flapping and lead-lagging modes are also identical with the ones given in [43] that the blade flapping modes are much farther away from the imaginary axis with respect to the blade lead-lagging modes and the magnitude of the frequency bound for the blade flapping modes is larger than the one for the blade lead-lagging modes (see Figure 2).

It is also required to note that all trim results obtained using our model also showed good correspondence with data given in the literature (see [31] for more details). For instance, the trim values for straight level flight at 40 kts were

(3) x40 kts0 =0.2753,0.0370,−0.0908,0.4857︸θ00,θc0,θs0,θT0,−0.0456,0.0272︸ϕA0,θA0,   0.0795,0.0592,0.0252,0︸β00,βc0,βs0,βd0,0.0218,0.0010,−0.0082,0︸ζ00,ζc0,ζs0,ζd0,   1.2523,6.2236,9.2217︸χ0,λ00,λc0T.

Here {*θ*_0_0__, *θ*_*c*_0__, *θ*_*s*_0__, *θ*_*T*_0__}, {*β*_0_0__, *β*_*c*_0__, *β*_*s*_0__, *β*_*d*_0__}, {*ζ*_0_0__, *ζ*_*c*_0__, *ζ*_*s*_0__, *ζ*_*d*_0__}, and {*ϕ*_*A*_0__, *θ*_*A*_0__} vectors are trim values of conventional helicopter controls, blade flapping angles, blade lead-lagging angles, and Euler angles, respectively, and all are given in unit of radians. The trim vector of linear downwash is {*χ*_0_, *λ*_0_0__, *λ*_*c*_0__} where *λ*_0_0__, *λ*_*c*_0__ are trims of collective and longitudinal cyclic downwash in m/s and *χ*_0_ is the trim of wake skew angle given in radians.

## 3. Illustration of MHT

**Figure 3:**
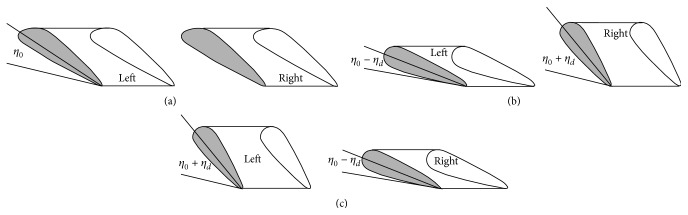
(a) Collective MHT angle, (b) left negative and right positive differential MHT angles, and (c) right negative and left positive differential MHT angles.

MHT angles (i.e., collective and differential) are illustrated in Figure 3. Collective motion refers to the movement of left and right horizontal tails in the same direction and magnitude simultaneously. On the other hand, differential motion refers to the movement of them in the opposite direction and the same magnitude simultaneously.

Angle of attack for left and right horizontal tails is calculated using

(4) $$\begin{array}{c} \alpha_{tp_{r}}=\alpha_{tp}+\eta_{0}+\eta_{d},\\ \alpha_{tp_{l}}=\alpha_{tp}+\eta_{0}-\eta_{d}, \end{array}$$

where *α*_*tp*_ is the angle of attack of classical fixed horizontal tail and *α*_*tp*_*r*__ and *α*_*tp*_*l*__ are angle of attack for moving right and left horizontal tails, respectively. Thelast MHT control (i.e., *l*_0_) is the control parameter of distance between helicopter center of gravity (cg) and horizontal tail (HT). The distance between cg and HT is found to be multiplying distance control parameter with the classical helicopter cg-HT distance.

## 4. Flight Control System (FCS)

For FCS, a variance constrained controller specifically output variance constrained control (OVC) is chosen. The OVC problem's description is given next.

For a given continuous linear time invariant (LTI) system

(5) $$\begin{array}{c} {\dot{x}}_{p}=A_{p}x_{p}+B_{p}u_{p}+w_{p},\;\;y=C_{p}x_{p},\;\;z=M_{p}x_{p}+v \end{array}$$

and a positive definite input penalty matrix *R* > 0, find a full order dynamic controller

(6) $$\begin{array}{c} {\dot{x}}_{c}=A_{c}x_{c}+Fz,\;\;u_{p}=Gx_{c} \end{array}$$

to solve the problem

(7) $$\begin{array}{c} \underset{A_{c},F,G}{\min}\;\;J=E_{\infty }u_{p}^{T}Ru_{p}=\mathrm{tr}\left(RG\Phi G^{T}\right) \end{array}$$

subject to

(8) $$\begin{array}{c} E_{\infty }y_{i}^{2}\leq \sigma_{i}^{2},\;i=1,\ldots ,n_{y}, \end{array}$$

where *z* represents sensor measurements, *w*_*p*_ and *v* are zero-mean uncorrelated Gaussian white noises with intensities *W* and *V*, respectively, *σ*_*i*_^2^ is the upper bound imposed on the *i*th output variance, and *n*_*y*_ is the number of outputs. The quantity *J* = *E*_*∞*_ *u*_*p*_^*T*^ *Ru*_*p*_ is referred to as the control energy (or cost) and Φ is the state covariance matrix computed using the OVC algorithm (see [44, 45]). Here *E*_*∞*_≜lim_*t*→*∞*_ *E* and *E* is the expectation operator. The solution to the OVC problem is obtained from a linear quadratic Gaussian (LQG) problem by choosing appropriately the output penalty *Q* > 0. Specifically, *Q* is dictated by the constraints imposed on the output variances (i.e., *σ*_*i*_^2^ in (8)) and it can be obtained using the iterative algorithm described in [44, 45]. After the algorithm converges and *Q* is found, the OVC parameters are computed using

(9) $$\begin{array}{c} A_{c}=A_{p}+B_{p}G-FM_{p},\\ F=XM_{p}^{T}V^{-1},\\ G=-R^{-1}B_{p}^{T}K, \end{array}$$

where *X* and *K* are obtained by solving the following two algebraic Riccati equations:

(10a) $$\begin{array}{c} 0=XA_{p}^{T}+A_{p}X-XM_{p}^{T}V^{-1}M_{p}X+W, \end{array}$$

(10b) $$\begin{array}{c} 0=KA_{p}+A_{p}^{T}K-KB_{p}R^{-1}B_{p}^{T}K+C_{p}^{T}QC_{p}. \end{array}$$

Clearly, compared to LQG where the penalties are selected ad hoc, OVC has the advantage that the penalty *Q* is selected such that output variance constraints are satisfied.

## 5. Trimming and Simultaneous MHT and FCS Design

Now it is required to define the simultaneous trimming and FCS design problem for the helicopter with MHT. This problem makes use of the extra number of trim unknowns (i.e., the 3 MHT control trims) and the ability to create helicopter linearized state-space models in terms of these MHT control trims. Let *x* = {*η*_0_, *η*_*d*_, *l*_0_} be the set of MHT control trims. The problem of finding optimum trim values for MHT controls can be obtained via changing the traditional OVC design problem summarized in Section 4 if the dependencies *A*_*p*_(*x*), *B*_*p*_(*x*) are considered. It is important to note that here *x* denotes the MHT controls trim values. During the control problem, formulation *u*_*p*_ represents perturbedcontrol vector and includes the MHT controls. The FCS energy in (7) and the expected values (i.e. *E*_*∞*_ *y*_*i*_^2^, *i* = 1,…, *n*_*y*_) in (8) are now function of these MHT control trims also, in addition of the control matrices (*A*_*c*_, *F*, *G*). Therefore, the following optimization problem is created:

(11) $$\begin{array}{c} \underset{A_{c},F,G,x}{\min}\;\;J=E_{\infty }u_{p}^{T}Ru_{p} \end{array}$$

subject to (5), (6), and (8). Furthermore, the components of *x* are constrained (i.e., *x*_*i*_min⁡__ ≤ *x*_*i*_ ≤ *x*_*i*_max⁡__, see Table 2). This new optimization problem is much more complicated than traditional OVC design and how to solve it is discussed next.

## 6. Simultaneous Perturbation Stochastic Approximation (SPSA)

The problem of finding the optimum values of the MHT control trims during the simultaneous trimming and FCS design problem summarized in Section 5 is much more difficult than the traditional OVC design due to the introduction of the additional MHT trim optimization variables and the associated constraints on them. Since there is complex dependency between *J* and expected values of outputs of interest, computation of their derivatives with respect to these variables is analytically impossible. This recommends the application of certain stochastic optimization techniques. In order to solve it, a stochastic optimization method, namely, SPSA, is chosen. This method was successfully used in similar complex constrained optimization problems (see [8, 11, 31, 41]) before. SPSA has many advantages. First, SPSA is inexpensive because it uses only two evaluations of the objective to estimate the gradient (see [40]). It is also successful in solving constrained optimization problems (see [8, 11, 31, 41, 46]). Moreover, under certain conditions (see [41]) strong convergence of SPSA was theoretically proved. Its short summary is given next.

Let *x* denote the vector of optimization variables. For the classical SPSA, if *x*_[*k*]_ is the estimate of *x* at *k*th iteration, then

(12) $$\begin{array}{c} x_{\left[k+1\right]}=x_{\left[k\right]}-a_{k}g_{\left[k\right]}, \end{array}$$

where

(13) $$\begin{array}{c} g_{\left[k\right]}={\left[\frac{\Gamma_{+}-\Gamma_{-}}{2d_{k}\Delta_{[k]1}}\cdots \frac{\Gamma_{+}-\Gamma_{-}}{2d_{k}\Delta_{[k]p}}\right]}^{T}, \end{array}$$

*a*_*k*_ and *d*_*k*_ are gain sequences, *g*_[*k*]_ is the estimate of the objective's gradient at *x*_[*k*]_, Δ_[*k*]_ ∈ *R* ^*p*^ is a vector of *p* mutually independent mean-zero random variables {Δ_[*k*]1_ ⋯ Δ_[*k*]*p*_} satisfying certain conditions (see [47, 48]), and Γ_+_ and Γ_−_ are estimates of the objective evaluated at *x*_[*k*]_ + *d*_*k*_Δ_[*k*]_ and *x*_[*k*]_ − *d*_*k*_Δ_[*k*]_, respectively. An adaptive algorithm considering the requirement that the optimization variables must be between lower and upper bounds was previously developed and combined with OVC to solve the simultaneous actively and passively morphing helicopter and FCS design problem (see [8, 11]). The adaptation is via the gain sequences, *a*_*k*_ and *d*_*k*_, and they are

(14) $$\begin{array}{c} a_{k}=\min\left\{\frac{a}{{\left(S+k\right)}^{\lambda }},0.95\;\underset{i}{\min}\left\{\min\left(\varphi_{l_{i}}\right),\min\left(\varphi_{u_{i}}\right)\right\}\right\},\\ d_{k}=\min\left\{\frac{d}{k^{\Theta }},0.95\;\underset{i}{\min}\left\{\min\left\{\vartheta_{l_{i}}\right\},\min\left\{\vartheta_{u_{i}}\right\}\right\}\right\}, \end{array}$$

where *ϑ*_*l*_ and *ϑ*_*u*_ are vectors whose components are (*x*_[*k*]*i*_ − *x*_min⁡_*i*__)/Δ_[*k*]*i*_ for each positive Δ_[*k*]*i*_ and (*x*_max⁡_*i*__ − *x*_[*k*]*i*_)/Δ_[*k*]*i*_ for each negative Δ_[*k*]*i*_, respectively. Similarly, *φ*_*l*_ and *φ*_*u*_ are vectors whose components are (*x*_[*k*]*i*_ − *x*_min⁡_*i*__)/*g*_[*k*]*i*_ for each positive *g*_[*k*]*i*_ and (*x*_[*k*]*i*_ − *x*_max⁡_*i*__)/*g*_[*k*]*i*_ for each negative *g*_[*k*]*i*_, respectively, and *d*, *a*, *λ*, Θ, and *S* are other SPSA parameters. The reader interested in the details of this algorithm is referred to [8, 11, 31].

In order to solve the simultaneous trimming and control design problem for optimal MHT trim values, the following algorithm is used in this paper.

**Step 1 .**

Set *k* = 1 and choose initial values for the optimization parameters, *x* = *x*_[*k*]_, and a specific flight condition (e.g., *V*_*A*_ = 40 kts straight level flight).

**Step 2 .**

Compute *A*_*p*_ and *B*_*p*_, design the corresponding OVC using (9), (10a), and (10b), and find the current value of the objective, Γ_*k*_ using (11); note that Γ_*k*_ = *J*_*k*_ for OVC.

**Step 3 .**

Perturb *x*_[*k*]_ to *x*_[*k*]_ + *d*_*k*_Δ_[*k*]_ and *x*_[*k*]_ − *d*_*k*_Δ_[*k*]_ and solve the corresponding OVC problems to find Γ_+_ and Γ_−_, respectively. Then compute the approximate gradient, *g*_[*k*]_, using (13) with *d*_*k*_ given by (14).

**Step 4 .**

If ‖*a*_*k*_ *g*_[*k*]_‖ < *δx*, where *a*_*k*_ is given by (14) and *δx* is the minimum allowed variation of *x*, or *k* + 1 is greater than the maximum number of iterations allowed, exit, else calculate the next estimate of *x*, *x*_[*k*+1]_, using *x*_[*k*+1]_ = *x*_[*k*]_ − *a*_*k*_ *g*_[*k*]_, set *k* = *k* + 1, and return to Step 2.

## 7. Results

### 7.1. Helicopter FCS Energy Saving

It is first required to note that, for all of the numerical results reported in this paper, the sensor measurements (*z* in (5)) were helicopter linear velocities, angular velocities, and Euler angles. The outputs of interest (*y* in (5)) were the helicopter Euler angles. The tolerance used for all of the OVC designs was 10^−7^. Firstly, the nonlinear helicopter model including MHT was trimmed using simultaneous trim and FCS design idea. The output variance constraints on helicopter Euler angles were $\sigma^{2}=10^{-4}\left[\begin{array}{ccc} 1 & 1 & 0.1 \end{array}\right]$ while the inputs of interest were all traditional helicopter controls (i.e., 3 main rotor controls and 1 tail rotor control) and the additional MHT controls (i.e., collective and differential control and distance control parameter). The helicopter FCS energy obtained after simultaneous trimming and FCS design is labeled as *J*_*r*_. Secondly, for the same flight condition, the same outputs and inputs of interest and the same constraints OVC were redesigned for the helicopter without MHT. The resulting helicopter FCS energy is labeled as *J*_*n*_. In order to see the benefits of using MHT on helicopters, the relative variation of the helicopter FCS energy, %*J*, was computed using %*J* = 100(*J*_*n*_ − *J*_*r*_)/*J*_*n*_.

**Figure 4:**
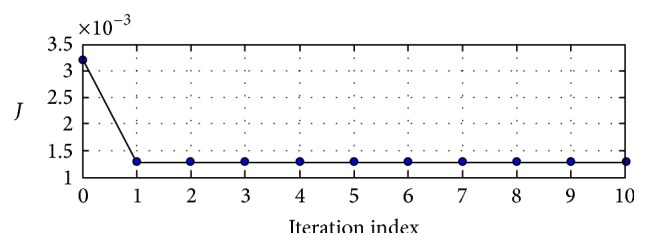
Cost optimization via SPSA.

The adaptive SPSA algorithm summarized in Section 6 was applied in order to solve the simultaneous trimming and design problem using the SPSA parameters of *S* = 5, *λ* = 0.602, *a* = 500, *d* = 20, and Θ = 0.101 via MATLAB software. For this design problem the algorithm was very effective in rapidly decreasing the helicopter FCS energy, *J*, converging quickly to a stable value, as seen in Figure 4 (see Table 2 for optimum MHT control trim values). Moreover, the FCS energy corresponding to the system obtained using simultaneous trimming and design was 59.4% lower than the FCS energy of system obtained using classical helicopter and traditional OVC (meaning that %*J* = 59.4%). The vector of trim values obtained after applying simultaneous trimming and design situation was

(15) x0 MHT40 kts=0.2830,0.0041,−0.1675,0.4826︸θ00,θc0,θs0,θT0,  −0.0620,0.070,︸ϕA0,θA00.0849,0.1348,−0.0027,0︸β00,βc0,βs0,βd0,  0.08634,−0.0014,−0.0123,0︸ζ00,ζc0,ζs0,ζd0,1.1942,6.6766,9.3000︸χ0,λ00,λc0T.

### 7.2. Closed Loop Simulations

In order to better evaluate the influence of MHT on helicopter performance, closed loop performance of classical helicopter and helicopter with MHT are compared. For this purpose, helicopter linearized state-space model obtained after simultaneous trimming and control design is used. For the discussions given next, closed loop system, which is obtained via integration of classical helicopter and OVC designed for it, is referred to as the 1st closed loop system. Similarly, closed loop system, which is obtained via integration of helicopter with MHT and OVC designed for it using simultaneous trimming and control design, is referred to as the 2nd closed loop system. In the figures given next, degrees are used to better show the behaviors of certain variables. The labels of classical and MHT are referring to the classical helicopter and helicopter with MHT, respectively. It is also significant to note that because linearized models are used, variables represent perturbations from their trim values in all the next set of figures.

**Figure 5:**
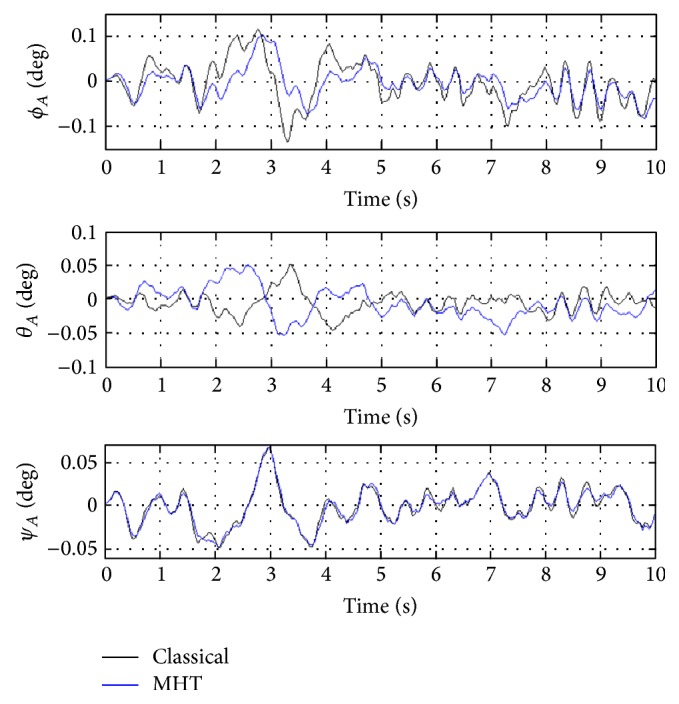
Responses of helicopter Euler angle states.

In Figure 5, closed loop responses of helicopter Euler angle states are given when the 1st closed loop system (solid black line) and 2nd closed loop system (solid blue line) are both excited by white noise perturbations. From Figure 3 it can be easily seen that, for both classical helicopter and helicopter with MHT, the qualitative (i.e., shape of the response) and quantitative (i.e., magnitude of the response) behaviors of Euler angles are basically the same. This can be explained using the fact that the expected values (*E*_*∞*_ *y*_*i*_^2^) of outputs of interest (i.e., helicopter Euler angles in this paper) are very close and satisfy the constraints (*E*_*∞*_ *y*_*i*_^2^ ≤ *σ*_*i*_^2^).

**Figure 6:**
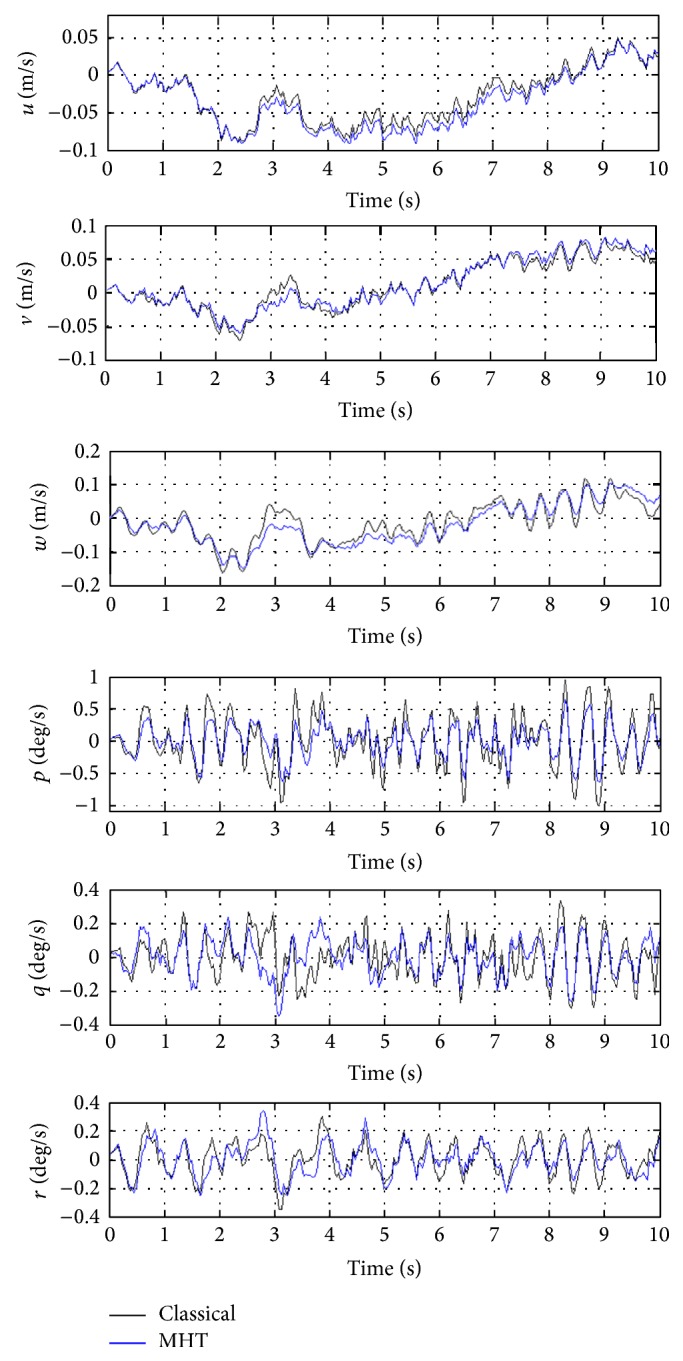
Responses of helicopter linear and angular velocity states.

In Figure 6, closed loop responses of helicopter linear and angular velocity states are given for the 1st closed loop system (solid black line) and 2nd closed loop system (solid blue line). Figure 6 shows that the linear and angular velocity states do not experience catastrophic behavior (meaning that fast and large variations do not occur). For both classical helicopter and helicopter with MHT, qualitative behaviors are similar. This nice behavior is clarified by the exponentially stabilizing effect of OVC (see [31] for more details).

**Figure 7:**
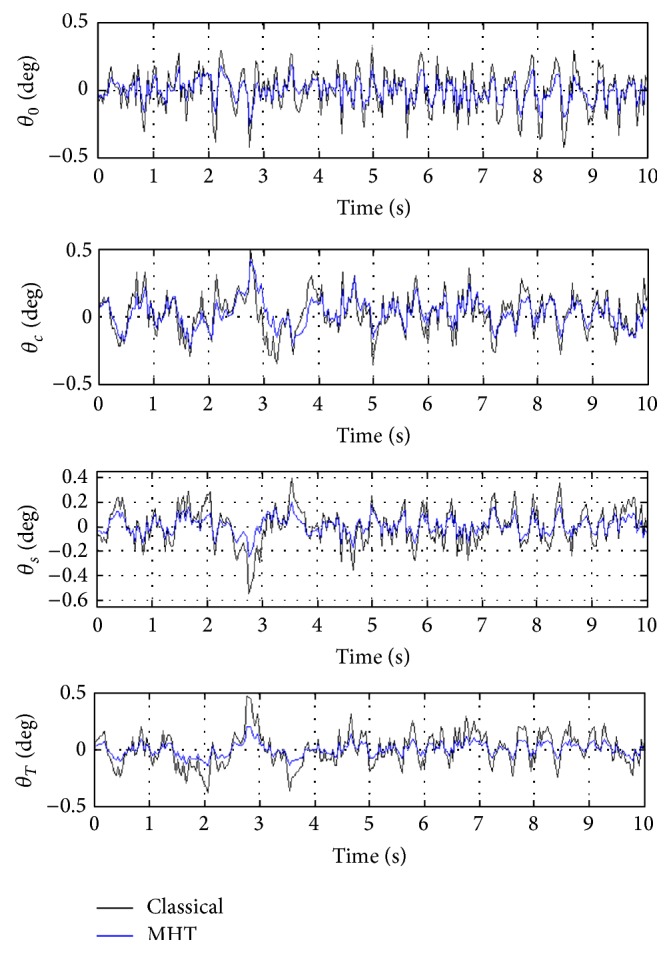
Responses of helicopter classical controls.

In Figure 7, closed loop responses of all traditional helicopter controls (i.e., 3 main rotor and 1 tail rotor controls) are given for both classical helicopter and helicopter with MHT. The most important observation related to the traditional helicopter controls is that there is substantial reduction in the peaks of the absolute values of these controls if MHT is utilized. It can be easily seen from this figure that lateral cyclic blade pitch angle (i.e., *θ*_*c*_) experiences with smallest reduction. Moreover, the control variations are smooth and small.

**Figure 8:**
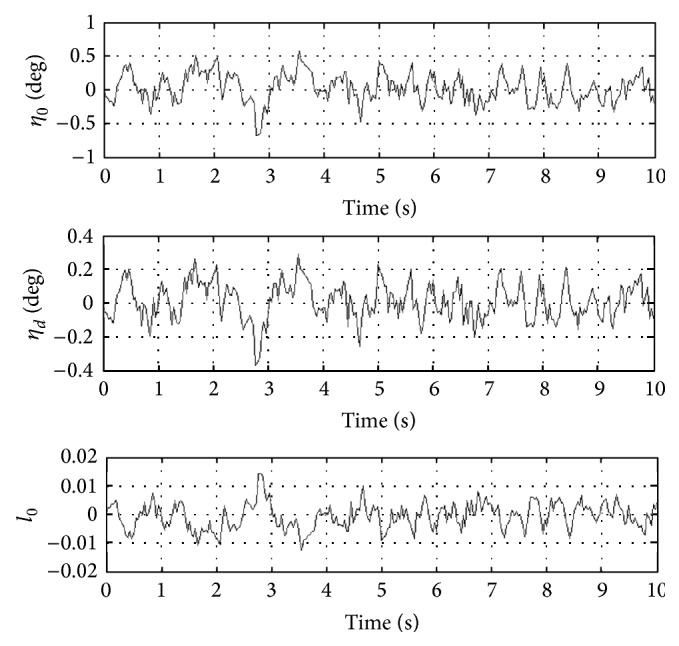
Responses of helicopter MHT controls.

In Figure 8, closed loop responses of MHT controls (i.e., collective and differential angles and distance control parameter) are given. It is clear from this figure that the peaks of the absolute values of all these additional controls are reasonable. Furthermore, they do not experience catastrophic behavior. Our extensive results also show that blade states do not experience catastrophic behavior and their qualitative behaviour is similar with/without MHT. This good behaviour is also clarified by the exponentially stabilizing effect of OVC.

## 8. Conclusions

Moving horizontal tail (MHT) idea is investigated in order to reduce helicopter flight control system (FCS) energy. Complex, control-oriented, physics-based nonlinear helicopter models are used for this purpose. Output variance constrained (OVC) controller is applied for helicopter FCS design. A stochastic optimization method is used in order to trim the helicopter during the simultaneous trimming and FCS design problem. Substantial FCS energy reduction (around 60%) is obtained using MHT. It is also important to note that this energy saving is obtained using small MHT control inputs. Nowadays such small changes are easily achievable and technologically feasible. It is also required to note that the FCS energy saving given in this paper is computed using linearized state-space model. In reality due to the nonlinearities it may be slightly different than this value.

Moreover, the qualitative behaviors of fuselage and blade states with/without MHT are similar and they do not display catastrophic behaviors. The outputs of interest (i.e., helicopter Euler angles) with/without MHT also display qualitatively and quantitatively similar behaviors while satisfying all of the output variance constraints. The peak values of traditional controls decrease with MHT clarifying the substantial reduction of FCS energy seen when MHT is applied.

## Figures and Tables

**Table 1:** Flight dynamics modes comparison.

Mode (rad/s)	*V*_*A*_ = hover		*V*_*A*_ = 40 kts		*V*_*A*_ = 80 kts	
	Padfield	Our model	Padfield	Our model	Padfield	Our model
1st	0.2772 ± 0.5008*i*	0.2215 ± 0.5966*i*	−0.1543 ± 0.9181*i*	−0.0434 ± 1.0846*i*	−0.1854 ± 1.0546*i*	−0.1736 ± 2.0642
2nd	−0.0410 ± 0.5691*i*	0.0587 ± 0.3589*i*	0.0275 ± 0.3185*i*	−0.0143 ± 0.3253*i*	−0.0085 ± 0.2074*i*	−0.0138 ± 0.1674*i*
3rd	−0.2697	−0.1449	−0.0976	−0.0703	−0.1358	−0.04786
4th	−0.3262	−1.1944	−0.9817	−0.7140	−1.5163	−0.7587
5th	−1.2990 ± 0.2020*i*	−0.6536 ± 0.3536*i*	−1.0394 ± 0.2798*i*	−0.6125 ± 1.1300*i*	−0.9252 ± 1.0503*i*	−0.6759 ± 1.8865

(Padfieldresults are taken from [43]).

**Table 2:** MHT parameters, constraints, and optimum points using SPSA.

MHT parameters	Nominal trim value	Lower bound Δ*x*_*i*_/*x*_*i*_	Upper bound Δ*x*_*i*_/*x*_*i*_	Optimum trim value	Change Δ*x*_*i*_/*x*_*i*_
*η*_0_	0 rad	−0.05	0.05	0.5022 rad	—
*η*_*d*_	0 rad	−0.05	0.05	−0.5037 rad	—
*l*_0_	3.80 m	−0.05	0.05	2.9317 m	−0.2285

## Nomenclature

*p*, *q*, *r*: Helicopter angular velocities, [rad/s]
*u*, *v*, *w*: Helicopter linear velocities, [m/s]
*ϕ*_*A*_, *θ*_*A*_, *ψ*_*A*_: Helicopter Euler angles, [rad]
*J*: Control energy, []
*l*_0_: Control parameter of distance between helicopter center of gravity and horizontal tail, []
*β*_0_, *β*_*c*_, *β*_*s*_: Collective and two cyclic blade flapping angles, [rad]
*ζ*_0_, *ζ*_*c*_, *ζ*_*s*_: Collective and two cyclic blade lagging angles, [rad]
*θ*_0_, *θ*_*c*_, *θ*_*s*_: Collective and two cyclic blade pitch angles, [rad]
*θ*_*T*_: Collective tail rotor angle, [rad]
*η*_0_, *η*_*d*_: Collective and differential moving horizontal tail angles, [rad]
FCS: Flight control system
MHT: Moving horizontal tail
SPSA: Simultaneous perturbation stochastic approximation.
